# Impact Of Environmental Variation On Host Performance Differs With Pathogen Identity: Implications For Host-Pathogen Interactions In A Changing Climate

**DOI:** 10.1038/srep15351

**Published:** 2015-10-19

**Authors:** Ikkei Shikano, Jenny S. Cory

**Affiliations:** 1 Department of Biological Sciences, Simon Fraser University, Burnaby, BC V5A1S6, Canada

## Abstract

Specialist and generalist pathogens may exert different costs on their hosts; thereby altering the way hosts cope with environmental variation. We examined how pathogen-challenge alters the environmental conditions that maximize host performance by simultaneously varying temperature and nutrition (protein to carbohydrate ratio; P:C) after exposure to two baculoviruses; one that is specific to the cabbage looper, *Trichoplusia ni* (TnSNPV) and another that has a broad host range (AcMNPV). Virus-challenged larvae performed better on more protein-biased diets, primarily due to higher survival, whereas unchallenged larvae performed best on a balanced diet. The environmental conditions that maximized host performance differed with virus identity because TnSNPV-challenge inflicted fitness costs (reduced pupal weight and prolonged development) whereas AcMNPV-challenge did not. The performance of TnSNPV-challenged larvae rose with increasing P:C across all temperatures, whereas temperature modulated the optimal P:C in AcMNPV-challenged larvae (slightly protein-biased at 16 °C to increasingly higher P:C as temperature increased). Increasing temperature reduced pupal size, but only at more balanced P:C ratios, indicating that nutrition moderates the temperature-size rule. Our findings highlight the complex environmental interactions that can alter host performance after exposure to pathogens, which could impact the role of entomopathogens as regulators of insect populations in a changing climate.

The harm a microorganism inflicts on its host is dependent on numerous factors, such that under some conditions it will act as a pathogen and in others not. Nowhere is this more evident than in the effects of malnutrition and climate warming on the changing prevalence or severity of some infectious diseases^1^,^2^. However, it is becoming increasingly clear that single factor experiments do not capture the complexity of this host-pathogen-environment interaction. There is a need to identify the interactive effects of nutritional change and altered climate, and how consistent these are across different pathogens, if we are to better predict future disease outbreaks.

In ectotherms, temperature and nutrition individually play powerful roles in host-pathogen interactions. They can have direct effects through thermal sensitivities and nutrient requirements of hosts and pathogens^3^,^4^, and indirect effects, through changes in host immune functioning, which generally increases with resource quality^5^,^6^ but varies unpredictably with temperature depending on the immune response measured^7^. Since multiple environmental factors are likely to change simultaneously in nature, knowing that immune functioning increases with resource quality may not provide a useful prediction if the magnitude of this effect varies with temperature. For example, in the Indian meal moth, *Plodia interpuctella*, the magnitude, and even the direction, of the individual effects of temperature, food quality and larval density on immunity were altered by complex interactions among the three factors^8^. Similarly, in the mosquito *Anopheles stephensi*, dietary supplementation to boost midgut immunity reduced infection by the rodent malaria parasite, *Plasmodium yoelii*, at intermediate temperatures but not at low and high temperature extremes^9^. Thus, complex interactions between environmental factors play an important role in the natural variation of pathogen virulence and host resistance. Many studies focus on immunological measures; however, in many systems the link between host susceptibility to a particular pathogen and a variety of immunological measures is not clear. There is therefore a need for more complex studies using ecologically relevant pathogens and their effects on host survival and other fitness traits.

To further complicate these interactions, hosts are often susceptible to a large number of diverse parasites^10^. These can differ in their mode of transmission, host specificity and tissue tropism, and consequently vary in the resistance mechanisms they trigger and the costs they inflict on their hosts^10^,^11^. For example, comparative studies have shown that specialist parasitoids^12^ and viruses^13^ triggered stronger immune responses in their insect hosts than their generalist counterparts, suggesting that they may impose greater survival costs. Therefore, not only is it important to understand how complex, multi-dimensional environments influence the outcome of host-pathogen interactions, we also need to discern whether results based on one pathogen can be extrapolated to another.

We examined the effects of temperature and dietary nutrients on the survival and development of the cabbage looper *Trichoplusia ni* (Hübner) after exposure to one of two ecologically relevant species of baculovirus, *Trichoplusia ni* single nucleopolyhedrovirus (TnSNPV) and *Autographa californica* multiple NPV (AcMNPV). Baculoviruses are highly pathogenic obligate killers that only infect the larval stage of susceptible insects. Transmission only occurs after the death of the infected larva. Both viruses occur in wild *T. ni* populations^14^,^15^, but TnSNPV is only known to infect *T. ni*^16^ while AcMNPV has a broad host range, infecting at least 15 lepidopteran families^17^.

Generalist insects such as *T. ni* can potentially feed on a vast array of plant species that can vary substantially in macronutrient content^18^. Thus, we established a nutritional gradient by varying the ratio of protein to carbohydrate (P:C ratio) in artificial diet^19^. The quality of food ingested throughout larval development alters host resistance to baculovirus challenge^20^,^21^,^22^, often through direct interactions between plant and pathogen in the midgut^23^. However, more recently, the quality of food ingested before or after baculovirus challenge have both been shown to alter the likelihood of survival^5^,^6^,^24^,^25^. The present study focused on the effects of P:C ratio and temperature post-pathogen challenge, since host resistance to baculoviruses increases with dietary P:C ratio consumed after infection^6^,^24^. Whether this effect is influenced by temperature and/or virus identity is not known. We hypothesized that virus challenge would alter host performance within the complex environmental space, and would differ depending on virus identity as each virus would inflict differing fitness costs. Since specialist pathogens may induce greater immune responses in their hosts^12^,^13^ and increasing P:C ratio enhances host immunity and survival after baculovirus-challenge^6^,^24^, we predicted that P:C ratio would more strongly influence host performance after TnSNPV challenge than after AcMNPV challenge. Temperature is not known to affect levels of baculovirus-induced mortality^26^,^27^. Therefore, the influence of temperature on insect performance may depend on its effects on development and interaction with dietary P:C ratio.

## Results

**Figure 1:**
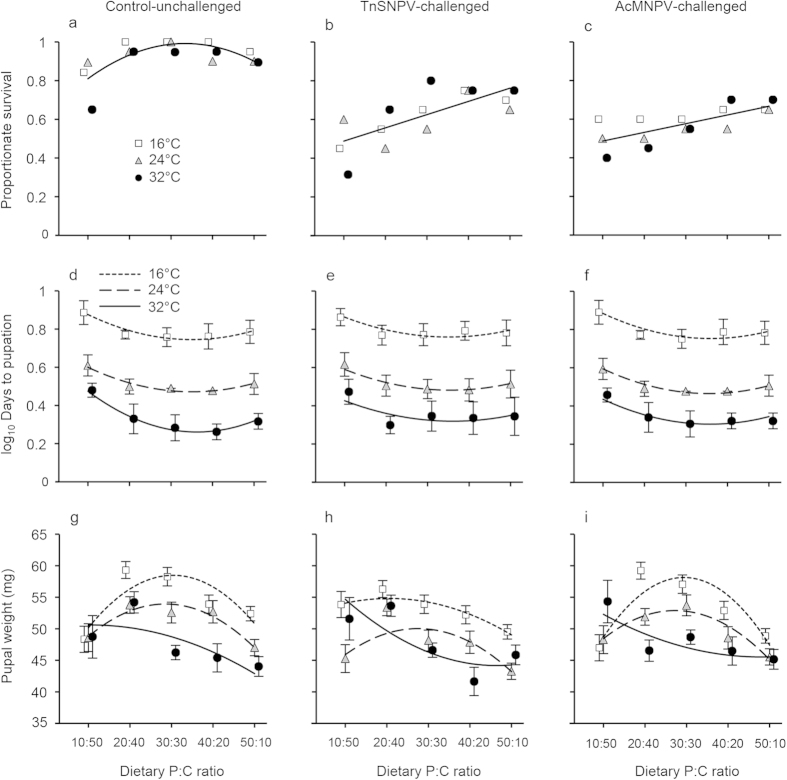
Impact of temperature and dietary P:C ratio on the survival, development time (log_10_ mean days to pupation ± SE) and dry pupal weight (mg ± SE ) of unchallenged control (a,d,g), TnSNPV-challenged (b,e,h) or AcMNPV-challenged (c,f,i) *Trichoplusia* *ni* in the fifth instar. Lines represent fitted minimal models (Table 2).

**Figure 2:**
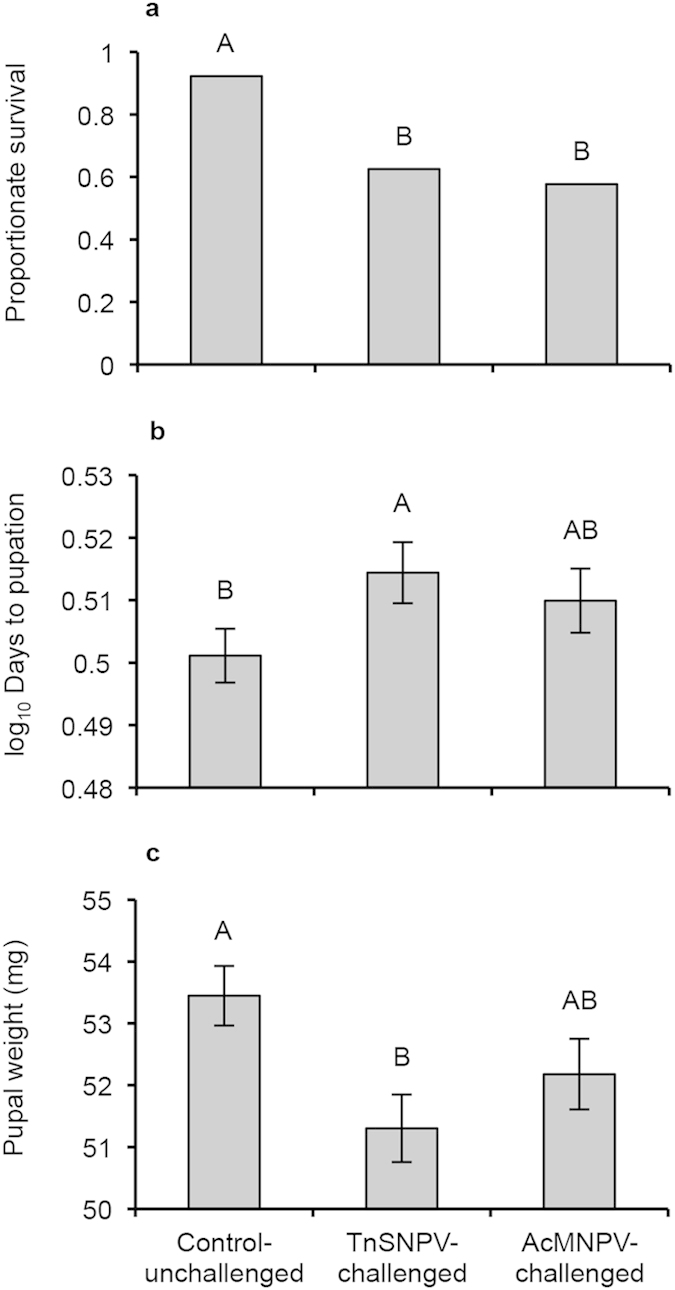
Fitness costs associated with virus challenge in fifth instar *Trichoplusia ni* measured by (**a**) the proportion of larvae survived, (**b**) development time (least squares mean log_10_ days to pupation ± SE) and (**c**) least squares mean dry pupal weight (mg). Different letters indicate significant differences (*P *< 0.05) identified by the main effect of virus treatment (no virus, TnSNPV challenge or AcMNPV challenge) from the GLM for survival (pairwise contrast; Table 1) and ANCOVA for development time and pupal weight (Tukey HSD comparison; Table 1).

Dietary P:C ratio affected survival after challenge by an equal dose of either TnSNPV or AcMNPV (Table 1). Temperature did not influence survival. While survival increased linearly with P:C ratio after both TnSNPV- and AcMNPV-challenge, survival increased more markedly after TnSNPV-challenge than AcMNPV-challenge (slope = 0.030 and 0.018 respectively; Fig. 1b,c and Table 2). Survival of unchallenged, control larvae decreased as the P:C ratio became increasingly unbalanced in both directions (Fig. 1a). These control deaths were due to dietary imbalance, and not contamination by virus since no viral OBs were observed under a phase contrast microscope. While virus challenge lowered survival relative to the control, the two viruses induced the same overall level of mortality (pairwise contrast: *X*^2^ = 2.79, *P* = 0.10; Fig. 2a and Table 1).

TnSNPV-challenged larvae took significantly longer to reach pupation compared to unchallenged-control larvae (Fig. 2b and Table 1). In contrast, development time of AcMNPV-challenged larvae did not differ significantly from unchallenged-control larvae. As expected, increasing temperature reduced development time (Fig. 1d–f and Table 2). Development time was non-linear across P:C ratios, and took longest on the most carbohydrate-biased diet.

Pupal weights of larvae challenged with TnSNPV were significantly lighter (4% lower than unchallenged control), while the pupal weight of AcMNPV-challenged insects did not differ significantly from unchallenged controls (Fig. 2c). P:C ratio had a non-linear effect on pupal weight that was modulated by temperature (Fig. 1g–i and Table 2). At 24 °C, pupal weight peaked on the balanced diet and declined as the P:C ratio became increasingly carbohydrate- or protein-rich. At 16 °C, pupal weights of control and AcMNPV-challenged larvae exhibited a similar response to P:C ratio as those at 24 °C, except that pupae were heavier at 16 °C on the balanced diets than at 24 °C. In contrast, pupal weights of TnSNPV-challenged larvae at 24 °C increased with dietary carbohydrate content and reached a plateau on the two most carbohydrate-biased diets. Pupal weights across P:C ratios at 32 °C differed from the responses at 16 and 24 °C such that pupal weight decreased non-linearly as the dietary P:C ratio increased. Pupal weight decreased with increasing temperature at balanced P:C ratios but not at the extremes.

**Figure 3:**
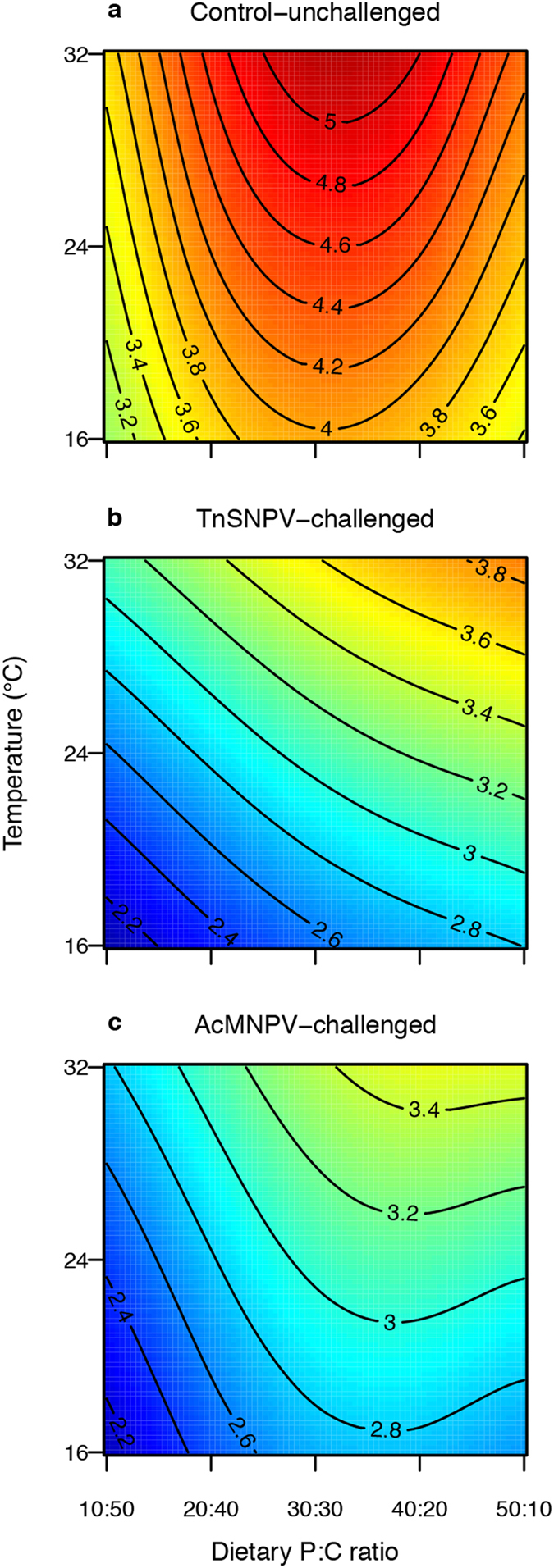
Response surface illustrating the bivariate effects of temperature and dietary P:C ratio on *Trichoplusia ni* performance after (a) no virus challenge, (b) TnSNPV challenge and (c) AcMNPV challenge. Performance was calculated by multiplying the rescaled values (1 to 2) for the individual probability of survival, dry pupal weight and the inverse of development time. Warmer colours and higher values on contour lines indicate higher performance (deep red, highest performance; deep blue, poorest performance).

The composite measure of performance, which was estimated at the individual level by multiplying an individual’s probability of survival, pupal weight and the inverse of development time, was influenced by a complex three-way interaction between P:C ratio, virus treatment (no virus, TnSNPV challenge or AcMNPV challenge) and temperature (Table 1). Performance of unchallenged larvae peaked on a relatively balanced diet at all temperatures, indicated by warmer colours on the response surface (Fig. 3). Performance of the survivors of TnSNPV challenge increased with P:C ratio at all temperatures, relative to unchallenged larvae. In contrast, temperature affected the performance of AcMNPV-challenged insects, such that at 16 °C, maximal performance was achieved on a slightly protein-biased diet, but as temperature increased, maximal performance was achieved on increasingly protein-biased diets.

## Discussion

Our results clearly demonstrate that the response to pathogen exposure can be influenced by environmental factors. Host survival can change depending on dietary constraints, and overall host performance can be modulated by the interaction between diet and temperature. In addition, we show that a host’s response to environmental variation differs with the identity of the infecting pathogen (Fig. 3). Maximal performance of unchallenged *T. ni* across temperatures occurred at a relatively balanced P:C ratio, similar to that selected when *T. ni* choose their own dietary nutrient ratios (1.3p:1c^28^). For *T. ni* challenged by the ‘specialist’ virus (TnSNPV), increasing survival with P:C ratio combined with a more moderate effect of P:C ratio on pupal weight compared to control larvae, caused performance to increase with dietary protein content across all temperatures. In contrast, challenge by the ‘generalist’ virus (AcMNPV) caused maximal performance to shift to a slightly more protein-biased P:C ratio at 16 °C in comparison to unchallenged control larvae, and as temperature increased, maximal performance was achieved on increasingly more protein-biased P:C ratios. The response of the AcMNPV-challenged larvae differed from those challenged by TnSNPV because the influence of diet on survival was less and pupal weight was more strongly influenced by the marked interaction between diet and temperature.

As we predicted, P:C ratio more strongly affected the survival of TnSNPV-challenged larvae, suggesting that the dietary boost in immune functioning or other resistance mechanisms was more effective against the specialist virus. In addition we found significant fitness costs (prolonged development time and reduced pupal weight) associated with surviving TnSNPV-challenge, but not AcMNPV. As baculoviruses are obligate-killers requiring host death for transmission and observable infection always results in death before the adult stage, we have taken any differences in host fitness traits as costs of surviving virus challenge, rather than a direct cost of virus infection (virulence). A key first line of defence against NPVs that may affect survival costs is the sloughing of infected midgut cells. Survival costs may also have resulted from an increased protein demand of immune responses to NPV^6^ and possible self-harm caused by immune responses, such as enzyme cascades (e.g. phenoloxidase) that lead to the activation of cytotoxic compounds that indiscriminately damage pathogens and host tissues^29^. An increase in mitochondrial generation of reactive oxygen species (ROS) associated with both increased protein consumption^30^ and higher respiration rate at higher temperatures^31^ may also inadvertently contribute to higher survival and survival costs after virus-challenge. ROS causes oxidative DNA damage, and hence, may inhibit the replication of baculoviruses, which are DNA viruses. ROS in the foliage of plants, when ingested together with baculoviruses, are known to inhibit baculoviral disease^32^. The compounding effects of increasing temperature and dietary protein on ROS production at 32 °C may also explain the decline in pupal weight with increasing dietary protein content.

The greater survival costs incurred by *T. ni* in response to TnSNPV-challenge compared to AcMNPV-challenge, suggests that it might be more costly for *T. ni* to fight TnSNPV challenge. *Spodoptera exigua* cells up-regulated more immune-related genes in response to infection by the specialist SeMNPV than by AcMNPV^13^. For *T. ni*, an increased response to TnSNPV challenge may be adaptive given that TnSNPV infection is more common in field-collected *T. ni* cadavers than AcMNPV^14^,^33^, which might be indicative of more frequent exposure to TnSNPV. Differential survival costs may also arise in response to the different infection strategies of the two viruses. The single nucleocapsid (SNPV) phenotype can establish more midgut infection foci compared to multiple NPV (MNPV), but is slower to infect tracheal cells and cause a systemic infection^34^. Thus, TnSNPV may induce higher rates and longer periods of midgut sloughing, which may cause greater impairment of nutrient absorption and a higher cost of replacing sloughed midgut cells. Although the host specificity of the viruses and the responses they elicit may play a role in the differential costs observed, many unknown differences exist between TnSNPV and AcMNPV. For example, the TnSNPV genome is 30 kb larger than AcMNPV^35^ and evolved resistance to TnSNPV in *T. ni* does not result in cross-resistance to AcMNPV^36^. Thus, more comparative studies on differential costs and virulence inflicted by other generalist and specialist viruses are needed to support our findings.

Some insects can alter their temperature through thermoregulatory behavior as a means to increase their ability to fight off pathogens. This research has focused primarily on behavioural fever, where infected locusts, grasshoppers and houseflies survive longer after fungal infections at warmer temperatures. When given the choice, infected insects will move to warmer areas of their environment^37^,^38^,^39^,^40^,^41^. However, cold temperature has also been shown to prolong the survival of bumblebee workers parasitized by parasitoid conopid flies^42^ and increase the survival of bacterially infected *Drosophila melanogaster*^43^. This bidirectional effect of temperature on host-pathogen interactions is likely due to a combination of differences in the thermal sensitivities of host and pathogen^3^ and the complex effect of temperature on insect immune functioning which can be positive or negative depending on the immune parameters measured^7^,^43^,^44^,^45^,^46^,^47^. As such, the ability of a host species to tolerate and resist pathogens at elevated temperatures can vary with pathogen identity. For example, the cricket *Gryllus texensis* is more resistant to the Gram-negative bacterium *Serratia marcescens* at an elevated temperature, but is more susceptible to the Gram-positive bacterium *Bacillus cereus* at temperatures below or above the average field temperature^44^. We found no effect of temperature on the survival of *T. ni* after AcMNPV or TnSNPV exposure, consistent with the thermal ecology of the western tent caterpillar and its NPV^26^ and the potato tuber moth and its granulovirus^27^. The temperature tolerance of our two viruses are likely high as exposure of the budded form of AcMNPV, which spreads infection between cells, to temperatures as high as 45 °C does not affect its infectivity^48^. Our findings also suggest that any temperature-induced changes in immune functioning after virus ingestion are not relevant for surviving baculovirus infection. We should note, however, that temperature can have profound influences on insect immune functioning^7^,^43^,^44^,^45^,^46^,^47^, development^49^,^50^ and behaviour^51^,^52^ that may predetermine susceptibility prior to baculovirus challenge.

Increasing rearing temperature reduces the adult body size of most ectotherms (temperature-size rule), potentially through thermal sensitivity of growth rates and smaller cell size^49^,^50^. A reversal of the slope of this thermal reaction norm (i.e. the pattern of phenotypic expression of a single genotype across a range of temperatures) was observed in *Manduca sexta* on a poor quality host plant^53^. Interestingly, *T. ni* followed the temperature-size rule at moderate P:C ratios but not at the extremes, indicating that dietary macronutrient ratios can moderate the temperature-size rule. Increased energy used at higher temperatures to fuel the increased basal metabolic rate associated with rapid growth can reduce lipid storage for energy^54^. This change in energy allocation might contribute to increasing pupal weights with dietary carbohydrate content at 32 °C, since there would be more energy from ingested carbohydrate to allocate to both metabolism and lipid storage^52^,^55^. Moderation of temperature-induced differences in growth rate has also been shown in response to plant allelochemicals added to artificial diet^56^ and reduced protein content of diet^57^, suggesting that the temperature-size rule may be limited to a restricted range of non-stressful environmental conditions^53^. Virus-challenge did not influence the temperature-size rule. However, given the stressful nature of fighting off pathogens and its effect on body size, further examination of the temperature-size rule in response to pathogens that inflict greater developmental costs are warranted.

Rising temperature from climate change is predicted to increase pathogen transmission and disease outbreak intensity in insect herbivores, as increased feeding rate would elevate the herbivore’s risk of encountering pathogens^51^. However, changes in innate susceptibility, the other side of the transmission process, are less clear. Our study indicates that baculovirus infection (the level of fatal infection incurred after pathogen ingestion) is not sensitive to temperature changes; however, the situation for other insect pathogens will depend on the insect-pathogen system. Nutrition and host plant quality are clearly very important for disease susceptibility and climate warming can also act indirectly by reducing host plant quality for the development of the herbivore^58^. Increasing temperature and CO_2_ associated with climate warming can increase plant defensive traits, such as leaf toughness and plant secondary compounds^59^, while at the same time decrease nitrogen content, thereby increasing the carbohydrate to nitrogen ratio^59^,^60^,^61^ (i.e. decreasing P:C ratio). Since *T. ni* survival and performance after virus-challenge are greater at higher dietary P:C ratios, the negative effects of pathogen-challenge will be exacerbated as the availability of dietary nitrogen decreases with climate warming. Moreover, larvae of another Noctuid moth, *Spodoptura litura*, have been shown to select a lower dietary P:C ratio at higher temperatures when given an opportunity to compose its own diet to meet higher energy demands^52^. Such nutritional changes in host plants and behavioural changes in nutrient intake are likely to increase susceptibility and compound the increased pathogen transmission risk resulting from increased feeding rate. As the effect of dietary P:C ratio on *T. ni* performance varies more with temperature after exposure to the generalist virus (AcMNPV) than the specialist (TnSNPV), it is clear that nutrition will be a key factor influencing host-pathogen interactions in a warming climate. This has implications for both the use of entomopathogens as biological control agents and more generally in terms of their regulation of natural insect populations. This study highlights a complex interplay of nutritional and temperature related effects on host performance and hence population density that may not be predictable from one pathogen to another.

## Methods

### Insects and viruses

The cabbage looper, *Trichoplusia ni*, (Lepidoptera: Noctuidae) is a widespread, generalist pest in many parts of North America, Europe, Asia and Africa, where it feeds primarily on crucifers but can also consume many other plant species. *T. ni* larvae were obtained from a laboratory colony that was originally collected from a commercial tomato greenhouse in British Columbia, Canada^62^. They have since been maintained at 25 °C and 16 h light and 8 h dark cycle on a wheat germ-based artificial diet^22^ which has a protein to carbohydrate ratio of approximately 1:1.1 (Bio-Serv, Frenchtown, NJ, USA). Prior to use in the experiment, *T. ni* were reared individually until the late fourth instar. Samples of TnSNPV isolate FV#34^33^ and the standard AcMNPV strain E2 were both amplified in *T. ni*. Viral occlusion bodies (OBs) were counted using an improved Neubauer brightline haemocytometer (0.1 mm deep; Hausser Scientific) at 400x magnification on a phase contrast microscope, and serial dilutions were made to produce the experimental doses.

### Treatment diets

Five isocaloric, chemically defined artificial diets were produced^55^ with protein to carbohydrate ratios (P:C) of 50:10, 40:20, 30:30, 20:40, 10:50. These ratios encompass a realistic range, as the protein and carbohydrate content in nine Brassicacae species, which are the preferred host plants of *T. ni*, contained 12–37% dry weight in protein (% nitrogen multiplied by conversion factor of 6.25) and 11–60% dry weight in digestible carbohydrate^63^. Supplementation of soil with nitrogen can increase protein content up to 50% in a Brassicacae species^64^.

### Experimental protocol

Moulting late 4^th^ instar larvae were allowed to complete their moult in individual wells of a 24-well cell culture plate (Corning Life Sciences, Tewksbury, MA, USA) without food. Freshly moulted, unfed final (5^th^) instar larvae were weighed, and then assigned to “virus treatment” groups consisting of a no virus control or virus challenge with AcMNPV or TnSNPV (300 larvae/virus treatment group). Larvae were fed a dose of 360 OBs (previously determined LD_50_ for both virus isolates; I. Shikano, unpublished data) of either AcMNPV or TnSNPV in 2 μl of distilled water applied to a 3 × 2 mm (length x diameter) diet plug or just water for controls. Each larva was left in individual wells of a 24-well cell culture plate with a single diet plug for 24 h at 25 °C to consume the entire diet plug. All larvae consumed the entire viral dose.

Larvae were then reared individually in 30 ml plastic cups (Solo Cup Company, Lake Forest, IL, USA) at one of three constant temperatures (16, 24, 32 °C) with a block of one of five treatment diets (~0.7 g). Thus, it was a 3 × 3 × 5 design with three virus treatments (TnSNPV, AcMNPV or no virus), three temperatures and five P:C ratio diets for a total of 45 treatment groups, and 20 larvae reared in individual cups per treatment group. Death or pupation was recorded every 12 h and diets were replaced every 24 h. Surviving pupae were collected immediately after the pupal case hardened and were placed in a drying oven at 50 °C for 48 h until constant mass (determined by weighing a sub-sample at intervals) and then weighed. Three larvae did not feed on the treatment diets and were excluded from analyses.

### Statistical analyses

The impact of virus treatment (no virus, TnSNPV or AcMNPV challenge), and environmental variation (dietary P:C ratio and temperature) on larval survival was analyzed using generalized linear models (GLM; binomial error, logit link). Pupal weight, development time and insect performance (see below) were analysed using analysis of covariance (ANCOVA) with temperature and virus treatment as categorical terms and diet (P:C) as both linear (diet) and quadratic (diet^2^) continuous terms. Survival, development time and pupal weight were also analysed separately for each virus treatment (no virus, TnSNPV-challenged and AcMNPV-challenged) to illustrate their individual contributions to the composite measure of performance. Sex (as binary numbers) and initial larval mass (mass immediately prior to virus-challenge) were included as covariates for all ANCOVA and GLM. Data were transformed where necessary. Non-significant interaction terms were removed sequentially to produce the final minimal model using JMP 10 (SAS Institute, Cary, NC, USA).

Performance was estimated at the individual level by multiplying an individual’s probability of survival (obtained from the final minimal GLM for each virus treatment), pupal weight and the inverse of development time^6^,^54^. Each measure (survival, pupal weight, development time) was re-scaled from 1 to 2 ([x − x_min_]/[x_max_ − x_min_] + 1) before calculating performance. Non-parametric thin plate splines were used to fit a three-dimensional response surface of the bivariate effects of temperature and diet on insect performance using the fields package in R (version 3.0.1)^54^,^65^.

## Additional Information

**How to cite this article**: Shikano, I. and Cory, J. S. Impact Of Environmental Variation On Host Performance Differs With Pathogen Identity: Implications For Host-Pathogen Interactions In A Changing Climate. *Sci. Rep.* **5**, 15351; doi: 10.1038/srep15351 (2015).

## Figures and Tables

**Table 1:** Analyses of the effects of virus treatment, temperature and dietary P:C ratio for host survival using GLM and development time, pupal weight and performance using ANCOVA.

effect	Survival		Development time			Pupal weight			Performance		
	*X*^*2*^	*P*	DF	*F*	*P*	DF	*F*	*P*	DF	*F*	*P*
initial weight	**5.38**	**0.02**	1,622	**47.39**	**<0.0001**	1, 620	**60.75**	**<0.0001**	1, 604	**196.86**	**<0.0001**
sex	0.42	0.52	1, 622	**62.60**	**<0.0001**	1, 620	**24.56**	**<0.0001**	1, 604	1.55	0.21
virus	**78.70**	**<0.0001**	2, 622	**3.22**	**0.04**	2, 620	**6.52**	**0.002**	2, 604	**382.33**	**<0.0001**
temp	1.72	0.42	2, 622	**3414.29**	**<0.0001**	2, 620	**55.54**	**<0.0001**	2, 604	**114.41**	**<0.0001**
diet	**15.19**	**<0.0001**	1, 622	**111.66**	**<0.0001**	1, 620	**50.67**	**<0.0001**	1, 604	**91.77**	**<0.0001**
diet^2^	**10.52**	**0.001**	1, 622	**233.77**	**<0.0001**	1, 620	**53.11**	**<0.0001**	1, 604	**190.23**	**<0.0001**
virus × temp	3.71	0.45	4, 614	1.77	0.13	4, 612	1.88	0.11	4, 604	**4.11**	**0.003**
virus × diet	1.08	0.58	2, 620	1.34	0.26	2, 618	0.48	0.62	2, 604	**14.03**	**<0.0001**
virus × diet^2^	**9.37**	**0.009**	2, 618	2.22	0.11	2, 616	2.83	0.06	2, 604	**28.62**	**<0.0001**
temp × diet	4.55	0.10	2, 622	**3.33**	**0.04**	2, 620	**3.10**	**0.05**	2, 604	1.21	0.30
temp × diet^2^	3.54	0.17	2, 612	1.47	0.23	2, 620	**14.72**	**<0.0001**	2, 604	0.59	0.55
virus × temp × diet	1.59	0.81	4, 608	1.36	0.25	4, 608	0.30	0.88	4, 604	0.72	0.58
virus × temp × diet^2^	3.80	0.43	4, 604	1.30	0.27	4, 604	1.71	0.15	4, 604	**4.24**	**0.002**

virus = virus treatment (no virus, TnSNPV challenge or AcMNPV challenge).

**Table 2:** Analyses of the effects of temperature and dietary P:C ratio for host survival using GLM and development time and pupal weight using ANCOVA.

effect	Survival		Development time			Pupal weight		
	*X*^*2*^	*P*	DF	*F*	*P*	DF	*F*	*P*
Control—unchallenged								
initial weight	1.73	0.19	1, 262	**32.23**	**<0.0001**	1, 262	**59.51**	**<0.0001**
sex	0.54	0.46	1, 262	**35.60**	**<0.0001**	1, 262	**7.87**	**0.005**
temperature	4.95	0.08	2, 262	**803.44**	**<0.0001**	2, 262	**24.81**	**<0.0001**
diet	2.43	0.12	1, 262	**100.73**	**<0.0001**	1, 262	**14.03**	**0.0002**
diet^2^	**11.88**	**0.0006**	1, 262	**152.18**	**<0.0001**	1, 262	**38.46**	**<0.0001**
temperature × diet	1.75	0.42	2, 262	**5.68**	**0.004**	2, 262	**3.27**	**0.04**
temperature × diet^2^	2.81	0.25	2, 262	**3.22**	**0.04**	2, 262	**3.58**	**0.03**
TnSNPV—challenged								
initial weight	0.59	0.44	1, 180	**10.60**	**0.001**	1, 176	**11.96**	**0.0007**
sex	1.46	0.23	1, 180	**15.14**	**0.0001**	1, 176	**12.80**	**0.0004**
temperature	0.77	0.68	2, 180	**716.95**	**<0.0001**	2, 176	**9.82**	**<0.0001**
diet	**12.18**	**0.0005**	1, 180	**9.80**	**0.002**	1, 176	**18.41**	**<0.0001**
diet^2^	1.67	0.20	1, 180	**33.11**	**<0.0001**	1, 176	2.21	0.14
temperature × diet	1.51	0.47	2, 178	0.53	0.59	2, 176	0.76	0.47
temperature × diet^2^	3.54	0.17	2, 176	0.65	0.52	2, 176	**3.68**	**0.03**
AcMNPV—challenged								
initial weight	**3.87**	**0.05**	1, 166	**9.02**	**0.003**	1, 162	**4.14**	**0.04**
sex	2.99	0.08	1, 166	**14.45**	**0.0002**	1, 162	**6.33**	**0.01**
temperature	1.00	0.61	2, 166	**1256.41**	**<0.0001**	2, 162	**23.33**	**<0.0001**
diet	**4.40**	**0.04**	1, 166	**33.42**	**<0.0001**	1, 162	**18.80**	**<0.0001**
diet^2^	0.02	0.89	1, 166	**77.58**	**<0.0001**	1, 162	**21.60**	**<0.0001**
temperature × diet	2.23	0.33	2, 164	0.99	0.37	2, 162	0.13	0.88
temperature × diet^2^	0.11	0.95	2, 162	0.12	0.89	2, 162	**11.39**	**<0.0001**

Analyses were conducted separately for each virus treatment group. Significant results are in bold font.
